# A Critical Review of Commercial Collagen-Based Scaffolds in Bone Regeneration: Functional Properties and Clinical Evidence from Infuse^®^ Bone Graft

**DOI:** 10.3390/jfb16090313

**Published:** 2025-08-29

**Authors:** Niki Karipidou, John Paul Muller Gorley, Chrysoula Katrilaka, Chris Manglaris, Anastasios Nektarios Tzavellas, Maria Pitou, Angeliki Cheva, Nikolaos Michailidis, Eleftherios E. Tsiridis, Theodora Choli-Papadopoulou, Amalia Aggeli

**Affiliations:** 1 Department of Chemical Engineering, Faculty of Engineering, Aristotle University of Thessaloniki, University Campus, 54124 Thessaloniki, Greece; karipidn@cheng.auth.gr (N.K.); gorley.jp@gmail.com (J.P.M.G.); ckatrilak@cheng.auth.gr (C.K.); cmanglaa@cheng.auth.gr (C.M.); 2 3rd Department of Orthopedics, School of Medicine, Aristotle University of Thessaloniki, University Campus, 54124 Thessaloniki, Greece; tzav_a@hotmail.com (A.N.T.); etsiridis@auth.gr (E.E.T.); 3 School of Chemistry, Aristotle University of Thessaloniki, University Campus, 54124 Thessaloniki, Greece; pitoumaria91@gmail.com (M.P.); tcholi@chem.auth.gr (T.C.-P.); 4 Department of Pathology, School of Medicine, Aristotle University of Thessaloniki, University Campus, 54124 Thessaloniki, Greece; antacheva@auth.gr; 5 Department of Mechanical Engineering, Faculty of Engineering, Aristotle University of Thessaloniki, University Campus, 54124 Thessaloniki, Greece; nmichail@meng.auth.gr

**Keywords:** Infuse^®^/InductOs^®^, recombinant human BMP-2 (rhBMP-2), collagen scaffold, bone regeneration

## Abstract

This review article provides a comprehensive evaluation of Infuse^®^ and InductOs^®^, two ground-breaking recombinant human Bone Morphogenetic Protein-2 (rhBMP-2)-based bone graft products, focusing on their tissue-level regenerative responses, clinical applications, and associated costs. Preclinical and clinical studies demonstrate that rhBMP-2 induces strong osteoinductive activity, effectively promoting mesenchymal stem cell differentiation and vascularized bone remodeling. While generally well-tolerated, these osteoinductive effects are dose-dependent, and excessive dosing or off-label use may result in adverse outcomes, such as ectopic bone formation or soft tissue inflammation. Histological and imaging analyses in craniofacial, orthopedic, and spinal fusion models confirm significant bone regeneration, positioning rhBMP-2 as a viable alternative to autologous grafts. Notably, advances in delivery systems and scaffold design have enhanced the stability, bioavailability, and targeted release of rhBMP-2, leading to improved fusion rates and reduced healing times in selected patient populations. These innovations, alongside its proven regenerative efficacy, underscore its potential to expand treatment options in cases where autografts are limited or unsuitable. However, the high initial cost, primarily driven by rhBMP-2, remains a critical limitation. Although some studies suggest overall treatment costs might be comparable to autografts when factoring in reduced complications and operative time, autografts often remain more cost-effective. Infuse^®^ has not substantially reduced the cost of bone regeneration and presents additional safety concerns due to the rapid (burst) release of growth factors and limited mechanical scaffold support. Despite representing a significant advancement in synthetic bone grafting, further innovation is essential to overcome limitations related to cost, mechanical properties, and controlled growth factor delivery.

## 1. Introduction

Restoring damaged or lost tissue is one of the most pressing challenges in modern medicine. This challenge is intensified by an aging global population and the increasing prevalence of degenerative diseases, traumatic injuries, and surgical interventions. There is a growing demand for effective solutions that go beyond symptom management and aim for true natural functional recovery. In this context, regenerative medicine in biomedical engineering has emerged as an interdisciplinary field at the forefront of medical innovation, focused on biologically inspired solutions for tissue’s repair and replacement.

Although often used interchangeably, *tissue engineering* and *tissue regeneration* are two key approaches that carry distinct conceptual and practical implications. In this review, *tissue engineering* involves inserting medical devices into defect sites to promote tissue’s maintenance, reconstruction, or healing [1,2]. *Tissue regeneration* implies regeneration using suitable medical devices that will eventually biodegrade and be entirely replaced by the new, healthy tissue, fully restoring the original architecture and function [3]. Integrating tissue templates that are foreign to the human body or to a specific tissue presents significant challenges. These include risks of immune rejection, infection, toxicity, pain, inflammation, and the need for additional surgical interventions [4,5,6,7,8]. Hence, ideal regenerative scaffolds should support cellular growth and differentiation while degrading harmlessly to prevent long-term complications.

Effective tissue regeneration relies on the integration level of three fundamental components: cells, growth factors, and scaffolds [9]. Cells provide the building blocks for new tissue, growth factors guide cell behavior and stimulate repair, and scaffolds offer a structure for cells to grow on. Depending on the regenerative context, the medical device must deliver the specific missing component(s), whether cellular, biochemical, or structural. Thus, an effective device design requires careful selection and optimization of each element—cellular components, signaling molecules, and scaffold—to suit the unique biological and mechanical environment of the targeted tissue [10]. Supporting tissue regeneration is like creating the right “home” for cells. This home must have the right inhabitants (cell types), be safe and compatible (biocompatibility), and provide the right signals (growth factors). Its “building materials” (scaffolds) must be strong yet degradable, with surfaces that guide cell behavior. Finally, the “layout” of the home—its porosity, pore size, and surface properties—must allow nutrient transport and angiogenesis. These complex demands make the design of such devices challenging [10,11,12,13,14].

**Figure 1:**
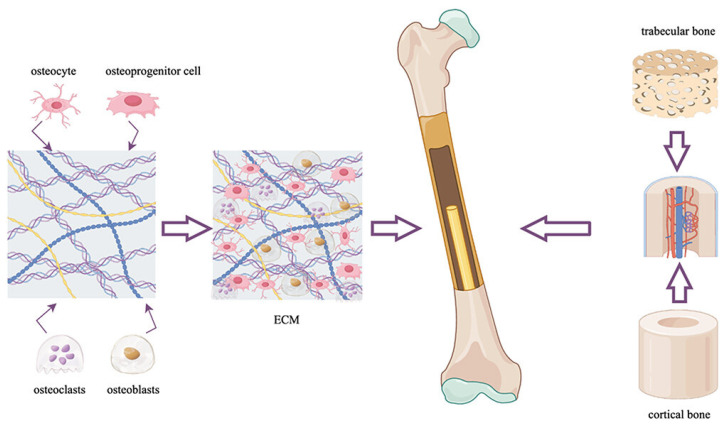
Schematic representation of bone tissue’s composition and regeneration. Bone consists of specialized cells (osteoprogenitor cells, osteoblasts, osteoclasts, and osteocytes) embedded in an extracellular matrix of collagen, hydroxyapatite, and water, which provides structural support and regulates signaling. Reprinted from Ref. [15].

Bone regeneration is an expanding field within tissue engineering, with considerable clinical potential. Bone itself is a dynamic, multifunctional organ with remarkable regenerative capabilities. In many cases, fractures can self-heal effectively with minimal medical intervention beyond proper alignment. This natural healing capacity is supported by a complex structure composed of various specialized cells, including osteoprogenitor cells, osteoblasts, osteoclasts, and osteocytes, as well as an extracellular matrix (ECM) composed of collagen, hydroxyapatite, and water. The bone’s ECM plays a central role in its function and regeneration. Acting as a structural scaffold, the ECM not only reinforces the mechanical strength of bone but also serves as a reservoir for signaling molecules essential for bone’s development, remodeling, and resorption (Figure 1) [15]. Skeletal remodeling continues throughout life, showcasing bone’s natural regenerative capacity. However, there are many cases of incomplete bone regeneration after damage, including in avascular necrosis, osteoporosis, and delayed union and non-union fractures, which often result from unstable fixation. Additionally, critical-size bone defects, which exceed the body’s innate regenerative threshold, resulting from trauma, infection, tumor resection, atrophy, and skeletal abnormalities can be too large for physiological bone regeneration to overcome and typically require surgical intervention for successful reconstruction [14,16]. Additionally, certain clinical contexts, such as spinal fusions performed to treat degenerative disc disease, necessitate bone formation in anatomically non-osseous regions, which the body cannot achieve without external support [17].

The current gold standard for inducing physiological bone formation is the autologous bone graft, bone harvested from the patient’s own skeleton, often the iliac crest, offering osteogenic, osteoinductive, and osteoconductive properties. Despite these advantages, autografting has notable limitations, including donor site morbidity, postoperative pain, increased surgical time and blood loss, and inconsistent quality and quantity of grafts [18]. To address these limitations, autografts are supplemented or replaced by allografts, bone sourced from cadavers or living donors, which lack viable osteogenic cells and may carry immunogenicity and disease transmission risks [19]. These challenges underscore a pressing, clinically necessary need for synthetic or engineered bone graft substitutes that are biocompatible, shelf-stable, and capable of recapitulating the essential functions of autografts without the associated morbidities [11]. Many researchers are currently attempting to design such next-generation devices [20,21,22].

Ideal bone regeneration devices must be osteoinductive, osteoconductive, and osseointegrative, meeting extensive physical, chemical, and biological requirements. *Osteoinduction* is a device’s ability to recruit progenitor cells and induce their osteoblastic differentiation, thereby initiating bone formation de novo, independent of the body’s natural bone formation processes. *Osteoconduction* relates to the scaffold’s three-dimensional architecture, which must support and guide the ingrowth of new bone tissue throughout its structure, and *osseointegration* is the formation of a fibrous tissue-free bony union between the implanted medical device and the host bone, ensuring mechanical stability and long-term functionality [14]. No commercially available device has yet achieved all three of these objectives consistently across clinical indications without causing significant side effects. Current solutions often involve trade-offs between efficacy and safety, highlighting the ongoing need for innovation in scaffold composition, structural design, and bioactivity to meet the complex biological and mechanical demands of bone regeneration.

There are many specific properties required for a device to be capable of osteoinduction, osteoconduction, and osseointegration, fulfilling a complex set of physical, chemical, and biological requirements. Firstly, one of the most critical challenges is that a bone regeneration device must have significant mechanical strength, because bone has an inherent load-bearing function. Without sufficient rigidity and stability, the scaffold cannot maintain its size and shape during regeneration. In addition, inadequate strength prevents cells from being exposed to the physical forces that stimulate their differentiation and activity [12]. Equally important is the promotion of vascularization, a prerequisite for sustained tissue growth and viability. However, achieving robust angiogenesis within bone is particularly challenging. As in most forms of tissue regeneration, the porosity and pore size of the scaffold material are crucial for cellularization, nutrient and waste transport, and angiogenesis. In bone regeneration, this presents a design dilemma: increasing porosity and pore size improves vascularization and cell migration, yet it simultaneously compromises the scaffold’s mechanical strength [8,12]. An ideal regeneration device must balance mechanical strength with the ability to support cell growth and blood vessels’ formation. Achieving this often requires innovative and carefully designed strategies. Another key factor is the synchronization of scaffold degradation with new tissue’s formation. With excessive degradation, regeneration may be incomplete, but with delayed degradation, an immune response on the part of the host becomes more likely [8]. Finally, retention and release of substances is also vital for a bone regeneration device. Water retention is linked to maintenance of scaffold volume and mechanical strength, as it allows a scaffold to absorb stress and withstand loading [23,24]. Release of substances, on the other hand, relates to the osteoinductive potential of a device. For reliable osteoinduction, the correct growth factors to promote cell migration, differentiation, and activity must be part of the device. The scaffold must have the proper affinity for the growth factors selected, to produce a slow, sustained, physiological release of the signal molecules and to help avoid side effects associated with growth factor leakage and burst release [11]. Alongside these bone-specific considerations, broader criteria relevant to all tissue engineering scaffolds remain essential. These include biocompatibility, biodegradability, thermal and surface properties, cost-effectiveness, and manufacturability—each of which influences the clinical utility and translational success of the device, turning its scientific discovery in the laboratory into a real-world medical treatment [10,11,12,13,14].

While no single device has yet met all the demanding criteria for ideal bone regeneration, significant advancements have been achieved. Among the most prominent developments is Medtronic’s Infuse^®^ Bone Graft, which has marked a major milestone in clinical translation in the bone regeneration field. Marketed as InductOs^®^ in Europe, Infuse is a porous collagen scaffold fabricated via freeze-drying and loaded with rhBMP-2, also known as dibotermin alfa, a highly potent osteoinductive growth factor [11]. Despite its widespread use and demonstrated efficacy in selected indications, Infuse has not consistently outperformed autografts and is associated with a range of adverse effects that raise concerns regarding its safety profile and limit its broader clinical adoption [25,26,27].

This review provides a critical and comprehensive overview of the current landscape of Infuse^®^, the leading commercial example of a collagen-based medical device for bone regeneration, to date in the field. Despite considerable progress in the development of collagen-based scaffolds for bone regeneration, comprehensive and consolidated evaluations remain limited, and detailed information—particularly regarding commercially available devices—is often difficult to access or scattered across proprietary and non-peer-reviewed sources. This review addresses this gap by providing a focused, critical analysis of Infuse’s structural characteristics, biological function, and clinical performance, explicitly integrating clinical studies, adverse-event profiles, and design–function relationships, as well as associated challenges. By doing so, both the potential and the limitations of collagen-based approaches are highlighted in the pursuit of next-generation bone regeneration therapies.

## 2. Composition Considerations and Commercial Advances

A diverse array of materials, including metals, ceramics, synthetic polymers, and biopolymers, have been explored for the fabrication of scaffolds in regenerative medicine [8,28,29,30]. Despite their widespread use, metals, ceramics, and synthetics can be cytotoxic, non-biodegradable, and cause immune responses, reducing their clinical applicability. Therefore, biopolymers have emerged as the preferred class of materials for bone regeneration devices. Among these, collagen stands out due to its abundance in the native extracellular matrix (ECM) and proven in vivo biocompatibility. Biocompatibility refers to the ability of a medical device to safely interact in vivo with living cells and tissue without causing harmful reactions in the long term. Collagen also provides controlled biodegradation and an intrinsic ability to support cellular attachment, migration, proliferation and differentiation [8,11,12,31,32]. Specifically in bone, the collagenous extracellular matrix is important for osteoblast adhesion and bone mineralization.

Nevertheless, the inherently weak mechanical properties of pure collagen impose significant challenges for its standalone application in the regeneration of load-bearing bone [8,11,33]. To address this, collagen is frequently enhanced via chemical cross-linking and other modification strategies. These are extremely important for fabricating devices that can endure compression and avoid later fracture, while maintaining porosity for vascularization and cell migration. Vascularization and cell migration are essential for osteoinduction, which is the process by which new bone formation is stimulated, and for overall tissue growth [11,12,25]. Collagen can be molded into various shapes and made extremely porous through different synthesis methods, making it permeable to vascularization and cell migration. As a result, many bone regeneration devices, including Infuse, are based upon a highly porous collagen scaffold, which attempts to provide satisfactory mechanical properties and allow for angiogenesis/cellularization, though fabrication of a scaffold with both of these properties remains challenging [11,12].

To overcome the limitations of current collagen carriers, next-generation scaffolds should be defined by quantitative engineering targets. Although unmodified FRESH-printed collagen exhibits a markedly low compressive modulus (~2.7 kPa), effective bone scaffolds require mechanically reinforced matrices capable of supporting at least 0.1–10 MPa (to approach cancellous bone stiffness) [34]. Architecturally, a pore size range of 100–400 μm (with additional microporosity) supports cell infiltration and vascular ingrowth; while these precise values in collagen remain to be experimentally validated, analogous bone scaffold reviews recommend these dimensions [35]. From a delivery perspective, BMP-2 binding affinity should target an apparent dissociation constant (Kd) in the range of ~10^−9^–10^−8^ M, consistent with affinities measured against glycosaminoglycans such as dermatan sulfate (Kd ≈ 2 × 10^−8^ M) and heparin (Kd ≈ 2.4 × 10^−9^ M) [36]. Together with cross-linking or composite reinforcement for enzymatic resistance and batch consistency, these criteria form a rational basis for the design of effective, next-generation collagen-based BMP carriers.

Among the commercially available collagen-based devices, Medtronic’s Infuse^®^ Bone Graft is especially interesting owing to its multiple regulatory approvals, robust clinical performance, and notably superior osteoinductive capacity relative to competing collagen-based grafts [25,37,38,39,40]. Alternative synthetic grafts are human-made materials designed to replace or support bone tissue that often combine polymers or biopolymers with inorganic components such as hydroxyapatite or calcium phosphate, to mimic the structure of natural bone (an approach often referred to as “void fillers”). Examples include Finceramica’s MaioRegen and RegenOss. These grafts have demonstrated a capacity to support bone regeneration predominantly via osteoconduction, guiding new bone growth along their structure. However, their osteoinductive potential remains inconsistent and often inadequate for reliable clinical outcomes due to the absence of incorporated osteoinductive growth factors. In contrast, Infuse distinguishes itself as the first commercially available osteoinductive autograft substitute, primarily attributed to its incorporation of rhBMP-2, a potent and well-characterized osteoinductive cytokine that effectively initiates de novo bone formation [41]. This highlights how Infuse^®^ actively induces bone formation rather than simply providing a framework for bone to grow on. This unique feature underscores Infuse’s pivotal role in advancing bone regeneration therapeutics, although its clinical application is tempered by considerations of safety, dosing, and cost.

## 3. Tissue-Level Response to rhBMP-2-Based Bone Grafts

The tissue-level response to rhBMP-2-based bone grafts, such as Infuse^®^ and InductOs^®^, is marked by a strong osteoinductive effect that triggers a cascade of regenerative events. RhBMP-2 is extensively used for its strong osteoinductive properties, particularly in the oral and maxillofacial region, where it enhances bone regeneration when combined with graft materials. Compared to autologous bone grafts, Infuse has demonstrated comparable or superior tissue-level healing outcomes, while avoiding donor site morbidity. However, the regenerative response depends heavily on dose and implantation site. Off-label use or excessive dosing can provoke exaggerated soft tissue reactions, particularly in sensitive areas like the cervical spine [41,42].

Upon implantation, rhBMP-2 recruits mesenchymal stem cells, which are early-stage cells found in bone marrow and other tissues that can develop into specialized cells such as bone, cartilage, or fat cells, and it induces their differentiation into osteoblasts, promoting rapid endochondral ossification. Histological studies reveal early deposition of bone matrix, increased alkaline phosphatase and osteocalcin levels, enhanced vascularization, and progressive remodeling into mature lamellar bone within weeks to months. The absorbable collagen sponge (ACS) carrier supports this process by providing a temporary scaffold that gradually resorbs, allowing seamless integration of newly formed bone into the host tissue [41,43,44].

Beyond promoting bone formation, rhBMP-2 also modulates the local immune microenvironment by transiently upregulating pro-inflammatory cytokines such as tumor necrosis factor-α (TNF-α), interleukin-1α (IL-1α), IL-1β, and IL-6. This early inflammatory response is thought to facilitate progenitor cell recruitment and tissue remodeling, processes critical for initiating bone healing. However, rhBMP-2’s application is not without risk.

**Figure 2:**
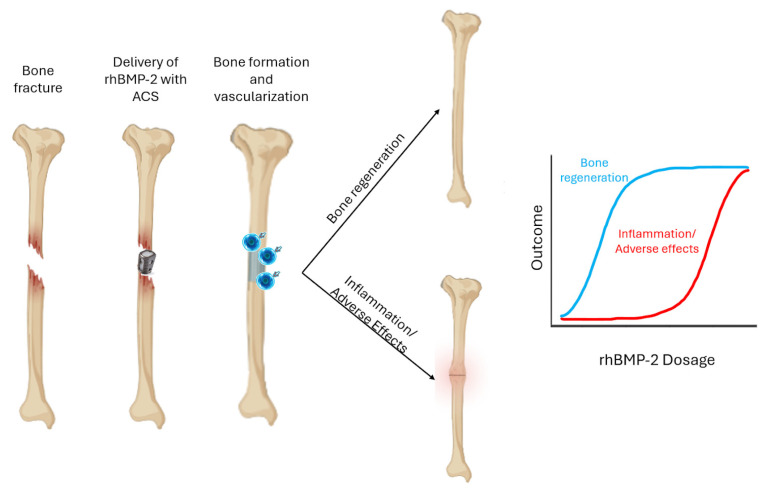
Schematic illustration of tissue-level response to rhBMP-2-based bone grafts.

Clinical and preclinical studies have documented significant adverse effects associated with rhBMP-2 use, including hematomas’ formation, inflammation, radiculitis, and heterotopic ossification, sometimes leading to neurologic impairment. These complications are particularly linked to the early burst release of higher-than-normal doses of rhBMP-2 from ACS, which can trigger excessive or prolonged inflammation. Additionally, rhBMP-2 has been shown to promote osteoclastogenesis, the formation and activation of osteoclasts, which are cells responsible for bone resorption, and increase osteoclast activity and bone resorption in a dose-dependent manner. Together, these findings highlight the delicate balance between the regenerative and inflammatory roles of rhBMP-2, emphasizing the need for controlled delivery strategies to minimize complications and optimize therapeutic outcomes [43,45]. The above points are schematically summarized in Figure 2.

## 4. Approved Clinical Applications

**Figure 3:**
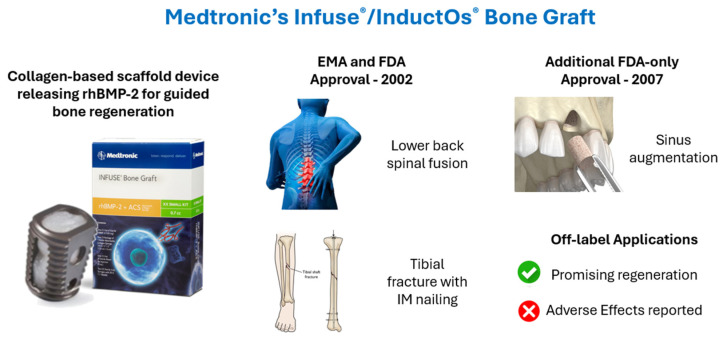
Medtronic’s Infuse/InductOs Bone Graft’s applications. Adapted from Ref. [47]. Adapted from Ref. [48].

The Infuse^®^ Bone Graft received EMA approval in Europe twice in 2002, under the name InductOs, for use in lower back spinal fusion and tibial fracture repair with intramedullary nailing, a surgical technique in which a metal rod is inserted into the marrow canal of the bone to stabilize fractures, representing two procedures with a high clinical demand and limited regenerative options [25,41,46]. It is also FDA-approved for these two indications, as well as an additional maxillofacial application. In this review, the device is referred to by its FDA-associated name, Infuse^®^ Bone Graft, given its broader regulatory approval in the United States [46]. These approvals have established Infuse^®^ as a pioneering product in the field of bone regeneration and a reference point for subsequent scaffold-based devices. Notably, Infuse^®^ has also been frequently employed in off-label clinical settings beyond its approved indications. While it has demonstrated promising regenerative outcomes in some of these off-label uses, it has also been associated with adverse effects [26,42]. Although such off-label applications are referenced when relevant, they fall outside the primary scope of this review and are not examined in detail. Figure 3 provides a schematic overview of Infuse^®^/InductOs’s applications, illustrating both approved indications and relevant off-label uses to facilitate a comprehensive understanding of the device’s clinical deployment.

### 4.1. Spinal Fusion

**Figure 4:**
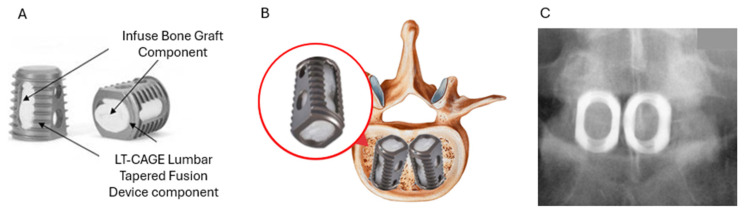
(**A**) The component and LT-Cage parts of the Infuse Bone Graft medical device. (**B**) Schematic illustration of the LT-Cages enclosing the Infuse Bone Graft in spinal fusion surgical procedure and (**C**) postoperative anteroposterior radiograph. Reprinted with permission from Ref. [25]. Copyright 2003 Lippincott Williams & Wilkins. Adapted from Ref. [53]. Adapted from Ref. [54].

Infuse received its first FDA approval in 2002, for single-level anterior spinal fusion to treat degenerative disc disease and spondylolisthesis up to grade one. During the procedure, the damaged disc is removed, and the collapsed space between the two vertebrae is expanded. After slight vertebral reaming, two of Medtronic’s tapered LT-Cages are placed in between the vertebrae with their larger ends oriented anteriorly, as illustrated in Figure 4B. The cages, illustrated in Figure 4A, have slots and holes to promote osseointegration on all sides except the smaller posterior end. They are then packed with the Infuse device, an absorbable Type I collagen sponge saturated with recombinant human Bone Morphogenetic Protein-2 (rhBMP-2) immediately prior to implantation. This approach facilitates localized delivery of rhBMP-2 at the fusion site, enhancing osteoinductive activity while minimizing the donor-site morbidity associated with autografts. A representative postoperative radiograph, shown in Figure 4C, demonstrates the positioning of the cages and graft material [25,49,50,51,52]. Overall, this combination of the device’s design and biologic augmentation underpins the reliable clinical outcomes observed with Infuse in spinal fusion procedures.

The pivotal clinical data supporting Infuse’s approval in this indication were drawn from pooled analyses of both published and unpublished studies, including the original investigational study submitted to the FDA [25,54,55,56]. The study included 679 patients divided in two cohorts, 277 treated with Infuse and 402 with autograft from the iliac crest, the conventional gold standard. Across most clinical endpoints, patients receiving Infuse demonstrated improved outcomes, including a modest but statistically significant increase in radiographic fusion rates at 24 months postoperatively. Infuse eliminated the need for autograft harvesting surgery, which was considered a major clinical advantage.

Based on these outcomes and the range of advantages summarized in Table 1, the original investigators concluded that Infuse represented a superior alternative to autograft and advocated for its adoption as the new standard of care for lumbar spinal fusion. At the time, no disadvantages were reported in their analysis, and there was a surge in both on- and off-label use of Infuse during the early years of its first FDA approval [37]. However, subsequent independent evaluations reported a range of adverse events, casting doubt on the initial claims and raising concerns about Infuse’s overall safety profile [18,26,37,57,58]. Most notably, the incidence of ectopic bone formation—believed to be a downstream effect of rhBMP-2’s diffusion—emerged as a frequent and clinically significant complication. These and other adverse effects associated with Infuse’s spinal application are summarized in Table 1 and underscore the need for further evaluation of its risk–benefit profile.

Adverse events associated with rhBMP-2 in spinal fusion have been reported with a wide range of frequencies across studies, often due to differences in definitions (radiographic vs. symptomatic), follow-up duration, and surgical approach. To harmonize these discrepancies, Table 2 collates the most consistent ranges from clinical trials, cohort studies, and systematic reviews. Radiographic heterotopic ossification (HO) is common (up to 75%), but symptomatic HO requiring intervention remains rare (≤2%). Other complications such as radiculitis, osteolysis, and seroma occur in a minority of patients, while events like incidental durotomy and retrograde ejaculation are largely approach-related rather than rhBMP-2-specific. To contextualize the clinical impact, the table also juxtaposes these adverse-event rates with typical absolute fusion gains at ≈24 months compared with iliac crest bone graft (ICBG). Across approaches, rhBMP-2 increases fusion success rates by approximately 5–15%, yet patient-reported outcomes show little or no consistent difference. This synthesis provides a balanced view of risks versus benefits, clarifying the net clinical effect of rhBMP-2 in spinal fusion.

Table 2 illustrates that although rhBMP-2 use in spinal fusion is associated with a spectrum of adverse events, most are infrequent, clinically mild, or approach-related rather than directly BMP-specific. Radiographic heterotopic ossification is common but rarely symptomatic, while complications such as seroma, infection, and radiculitis occur in only a minority of patients. Incidental durotomy and retrograde ejaculation reflect surgical exposure rather than intrinsic BMP toxicity. Importantly, when these risks are juxtaposed with the typical absolute gains in fusion success at ≈24 months (≈5–15% versus iliac crest bone graft), the overall balance suggests that rhBMP-2 provides a measurable technical benefit in achieving solid arthrodesis. However, since improvements in patient-reported outcomes are not consistently demonstrated, careful patient selection and procedural context remain critical.

The potential association between rhBMP-2 and cancer has been a matter of debate. Earlier concerns [66] suggested a possible dose-related signal, but independent re-analyses and subsequent meta-analyses of randomized trials and large observational cohorts [67,68,69,70] have found no statistically significant increase in absolute cancer risk. Across studies with a follow-up ranging from 2 to 7 years, the absolute risk difference between rhBMP-2 and controls was consistently <1%. Importantly, recent meta-analyses specifically addressed confounding by indication in observational studies, and no consistent evidence of excess cancer incidence was found. Current evidence therefore suggests no clinically relevant increase in malignancy risk after spinal use of rhBMP-2, although a longer follow-up remains warranted.

Ectopic bone formation and other adverse events are largely attributed to leakage and burst release of the massive rhBMP-2 dose contained in an Infuse device [11,42]. Prior to implantation, the collagen sponge is soaked with a 1.5 mg/mL solution of rhBMP-2 [57], resulting in an average dose of 6.2 mg per spinal level [37], far exceeding the estimated total physiological BMP-2 content in the human body (~2 mg) [59].

Unfortunately, this high dose is not retained effectively by the collagen scaffold, leading instead to a burst release profile with limited control. Very high local concentrations of rhBMP-2—well above what the body would naturally produce—at the implantation site have been called the single most contributory factor to most adverse events, including inflammation, osteolysis, and unintended osteogenesis at ectopic sites. Despite efforts to reduce the rhBMP-2 concentration—most commonly to 0.75 mg/mL—clinical outcomes consistently demonstrate diminished bone formation relative to the 1.5 mg/mL standard, even though the lower dose remains well above physiological levels [57]. The necessity of such high-concentration dosing may be partly explained by the limited binding affinity between rhBMP-2 and the collagen matrix, leading to rapid clearance and reduced bioavailability at the target site [11,42].

Furthermore, high concentrations of exogenous BMP-2 are known to induce expression of the endogenous BMP antagonist Noggin, which in turn suppresses BMP-2 signaling and may necessitate further dose escalation to overcome this feedback inhibition. Noggin’s resistance to inhibitors means limited therapeutic success in reducing its interference with rhBMP-2 [59]. Considering BMP-2’s effects extend beyond osteoblast differentiation and activity—including an influence on adipogenesis, inflammation, and osteoclast activation—side effects are expected if dosage and release cannot be properly controlled [57]. Given these limitations, an optimal delivery system for rhBMP-2 would ensure sustained, localized release within therapeutic windows, minimizing systemic exposure and mitigating the induction of negative feedback loops such as Noggin’s upregulation. In light of this, significant research has focused on developing advanced delivery platforms that enable controlled release kinetics and improved growth factor retention [71,72].

A separate group of adverse events less related to rhBMP-2 involves the device’s subsidence and dislodgement. As with any foreign implant, the use of the metal LT-Cage increases risks, including infection, inflammation, and pain related to the procedure [4,5,6,7,8]. The cage, designed for mechanical strength and bone graft fixation, can subside or dislodge, especially when excessive bone resorption occurs near the implant due to rhBMP-2-induced osteoclast activity [57], decreasing osseointegration between the cage and the spine. Despite these drawbacks, the spinal approval of Infuse created an extremely valuable alternative to autograft for lumbar fusion patients and represents a major step in the field of collagen-based bone regeneration.

### 4.2. Open Tibial Fracture

Infuse was FDA-approved for the second time in 2004 for use with intramedullary (IM) nailing in open tibial fracture repair [41]. Prior to approval, the open tibial fracture patient population was identified as a group that might benefit much from Infuse, as nonunion, a condition in which fractured bones fail to heal properly, and related complications are common [73]. For example, one study showed that 41% of open tibial fracture patients treated with IM nails, the most effective fixation device used in open tibial surgery, require a second surgical intervention [41,74]. Importantly, open tibial fractures differ from other applications of Infuse. In the other two clinically approved applications, there are some generally accepted optimal surgical practices and even a gold standard for bone graft material (autograft) [25,75]. In open tibial fracture, however, injury repair and tissue reconstruction is managed on a case-by-case basis, as clinical data on the subject is contradictory, and optimal practices differ based on bone defect size, soft tissue damage, and other complicating factors [74]. Since its approval for this application, Infuse represents one treatment option for open tibial fracture patients, but it is not necessarily vying for the gold-standard spot as is the case in other applications. In fact, standard care often does not include bone grafting, though bone grafting is gaining popularity in these procedures [27,73,74,76]. The most common form of bone grafting in open tibial fracture is autograft to treat large bone gaps, and grafting is often performed in a secondary intervention after initial fixation has failed to induce healing of fractures [77].

In the pivotal study of Infuse in this application (carried out by the BMP-2 Evaluation in Surgery for Tibial Trauma (BESTT) Study Group), 450 patients were split into three groups: a control consisting of standard care only (soft tissue management and IM nailing), a group with standard care plus Infuse at a reduced dose of 0.75 mg/mL rhBMP-2 (total dose of 6 mg), and a group with standard care and Infuse at the regular 1.5 mg/mL concentration of rhBMP-2. This study included patients with varying sizes of bone defect and different injury classifications, and patient assignments were stratified to include both lower and higher injury classifications in each treatment group. The general surgical procedure for fixation with IM nail consists of inserting the nail into the medullary cavity, locking it with screws to prevent rotation, and closing the wound [78]. In this study, grafting with Infuse was performed during the primary intervention, after fixation and before wound closure [73,79]. Importantly, compared with the control group, the 1.5 mg/mL concentration of rhBMP-2 group had a 44% reduced risk of intervention failure (defined as delayed union resulting in secondary intervention). Additionally, the 1.5 mg/mL group had significantly improved results in invasiveness of secondary intervention, rate of fracture and wound healing, and number of hardware failures. (For patients with higher injury classifications specifically, the 1.5 mg/mL rhBMP-2 group also had fewer infections.) The concentration-dependent improvements in the Infuse group are consistent with dosage studies across the three approved applications of Infuse and again suggest a burst release of rhBMP-2 from the collagen scaffold [11,73]. There were no reports of adverse events related to Infuse use, and researchers specifically mentioned that no increases in local soft-tissue calcification or remote ectopic bone formation were observed in patients treated with rhBMP-2 [73]. Results from this study showed the potential for Infuse to greatly improve outcomes for open tibial fracture patients by promoting healing, reducing complications, and removing any harvesting surgery necessary for autografting. The FDA’s approval for Infuse in this application followed, and rhBMP-2 is now used clinically for some open tibial fractures [27,73,76,80]. However, it is important to note that this initial study compared Infuse with a control group consisting of only fixation and wound care, without any bone regeneration implant.

A later study by the Major Extremity Trauma Research Center (METRC) split 30 large-size bone defect open tibial fracture patients into autograft and Infuse plus allograft groups for delayed (secondary intervention) bone grafting 6–16 weeks after initial open tibial fracture treatment with IM nailing. The autograft group had higher rates of fusion and clinical healing, lower musculoskeletal dysfunction scores, and fewer complications [27]. This study suggested that autograft and Infuse/allograft treatment in open tibial fractures with large size defects are not equivalent treatments, though it could not conclude superiority of either treatment because of the small sample size. This result differed from a previous study by Jones et al., which also split 30 patients into autograft and Infuse/allograft groups. The Jones study reported fracture healing without further intervention to be 20% higher in the Infuse/allograft group and concluded that Infuse/allograft was not inferior to autograft treatment [81]. The Jones study, however, included patients with closed and open fractures, as well as fixation via plates, external fixators, and IM nails, differing from the METRC study on only IM nail fixation of open fractures. Differences between these BESTT, METRC, and Jones studies include the use of autograft control, sample size, bone defect size, time of bone graft intervention relative to fracture, fixation method, and more, making it difficult to compare results. Taken together, these studies provide a broad picture of the current state of the literature on the use of Infuse in open tibial fracture. Treatment varies widely depending on the case at hand, and many complicating variables and contradictory studies have made it difficult to synthesize a large body of meaningful data on Infuse in open tibial fracture [74]. A side effect profile has yet to appear for this Infuse application, likely due to the lack of studies and quantitative data.

To summarize, Infuse in open tibial fractures improves clinical outcomes when compared with standard, graft-free treatment [73], but this result may be dependent on one or more of the many complicating factors involved in treating open tibial fractures, including defect size, reamed versus unreamed intramedullary nailing, time of grafting intervention, or others [27,76,82]. Further studies comparing Infuse with autograft and no graft treatment are warranted. In this application, Infuse may not achieve all desired regenerative outcomes, as its regenerative effects may be inferior to autograft, and the extremely high concentration of rhBMP-2 necessary for regeneration suggests suboptimal release of the growth factor [11,59,73]. However, the success Infuse has shown in this extremely complex and high-risk application is clinically relevant and an important advancement in collagen-based bone regeneration.

### 4.3. Maxillary Augmentation

Infuse^®^ Bone Graft received its most recent FDA approval in 2007 for sinus augmentation and localized alveolar ridge augmentation related to extraction socket issues [41]. As in lumbar fusion, Infuse is used in maxillary augmentation as a substitute for autograft bone [75]. Its approval was based on clinical evidence demonstrating that Infuse could promote high-density bone regeneration in the posterior maxilla, where bone volume is often insufficient for dental implant placement [83].

Sinus augmentation is a surgical procedure that increases bone height in the upper jaw (maxilla) beneath the sinus cavity, typically to enable placement of dental implants in patients with inadequate bone mass. Alveolar ridge augmentation refers to reconstructing the bony ridge that supports the teeth, particularly after tooth loss, to restore adequate bone volume for implant stability. Both are used in cases where jawbone volume is insufficient for conventional implantation [84].

**Figure 5:**
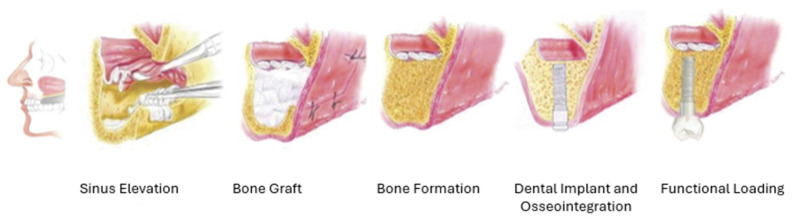
Schematic illustration of the maxillary sinus floor augmentation enclosing the Infuse Bone Graft component and implant placement. Reprinted with permission from Ref. [83]. Copyright 2009 Elsevier.

The process consists of surgically opening the gums, placing bone graft material on top of, beneath, or around the maxilla, and then resealing the gums. Depending on the patient’s original maxillary bone height, dental implants may be placed in the same operation or after allowing for bone regeneration, as presented in Figure 5 [75,85]. Usually, the only graft fixation or wound sealing devices used are resorbable collagen membranes [85], though in rare cases of a severe bone defect, a wire mesh may be used [86].

Although initial studies reported high success rates with minimal adverse events, post-approval surveillance identified safety concerns. The most frequently reported events in on-label maxillofacial applications during the first four years after approval were localized swelling (edema), erythema (redness), and pain. Within the same four years, there were additional reports to the FDA MAUDE Database of infections, wound complications, graft failure, hardware complications, tumor formation, and several other issues [42,83,87]. Approximately two-thirds of these reports were related to off-label craniofacial application of Infuse, but at least two reports of most adverse events were in on-label sinus or alveolar ridge augmentations. As in other Infuse applications, burst release of rhBMP-2 due to flawed binding between the porous collagen scaffold and rhBMP-2 is likely the root cause of most side effects in the maxillofacial application [11,42]. In one sinus augmentation study, the common side effect of edema was found to be dose-dependent, supporting the conclusion that a high localized concentration is the source of major and concerning Infuse side effects [42]. In general, the relatively low number (83) of adverse events reported in reference to Infuse maxillofacial operations within four years is mostly encouraging, considering that within the same four-year period, 846 adverse events regarding the alternate applications of Infuse (likely mostly spinal) were reported to MAUDE [87].

Several reasons may be suggested for the low incidence of side effects in on-label maxillofacial Infuse applications. Firstly, only substances native to bone tissue are implanted (the collagen scaffold, rhBMP-2, and possibly a collagen membrane for containment). In these maxillary augmentations, the scaffold does not support a load, as dental implants are only placed immediately if there is already enough native bone to anchor them [75,85]. Therefore, no fixation device or supporting cage is necessary, and no substance foreign to the human body is introduced. This minimizes many risks, including infection, toxicity, immune rejection, implant dislodgement, pain, and repeated surgery [4,5,6,7,8]. Additionally, though the concentration of rhBMP-2 is constant at 1.5 mg/mL for each of its three approved applications [57,79], the volume of bone graft used may differ between applications. In sinus augmentation, for example, the mean volume of any bone graft used to achieve 13.4 mm of maxillary bone height (more than sufficient for dental implantation) is 2.65 mL [88,89]. Relating this to the 1.5 mg/mL rhBMP-2 concentration of Infuse, the total dose of rhBMP-2 for each side of the mouth in sinus augmentation may be approximately 3.75 mg. While still much higher than physiological levels of BMP-2, a 3.75 mg dose is much lower than the reported average dose of rhBMP-2 used in the spinal application of Infuse (6.2 mg per spinal level) [37]. While the rhBMP-2 dosage is trending down across its applications, the lower dose used in oral procedures probably contributes to the lower incidence of majorly concerning side effects [87]. Additional factors that may contribute to the reduced side effect profile of Infuse in this application are the anatomical location and the reduced surgical complexity when compared with spinal fusion. In spinal fusion, especially in off-label applications at the narrower regions of the spine, the Infuse scaffold and its accompanying massive dose of rhBMP-2 are placed in close proximity to the spinal cord, and major complications can result if rhBMP-2 leaks and causes ectopic bone formation with subsequent spinal cord compression [57]. In sinus or alveolar ridge augmentation, however, a slight leakage of rhBMP-2 might be less symptomatic, as suggested by the lack of widely reported bone growth in abnormal locations (ectopic bone formation) in these Infuse applications.

In summary, the maxillary applications of Infuse perhaps represent one of the most promising collagen-based approaches currently available for achieving bone regeneration without autograft harvest, though outcomes can vary depending on defect type, surgical technique, and patient-specific factors. The high success rate and relatively low incidence and danger of side effects make Infuse a compelling clinical option for sinus or alveolar ridge augmentation. However, side effects do exist. An ideal bone regeneration device is yet to be found.

## 5. Cost

Costs of autograft bone treatments are high, especially because autografting requires increased operating times and hospital stays. A major aim of synthetic bone grafts is to reduce bone grafting costs for patients and hospitals [25,90]. Infuse costs between USD 2500 and USD 6000 per package, as it is summarized in Table 3 [52,54], a high price due to its incorporation of rhBMP-2. It is notable that this protein’s cost has not decreased since it became available over twenty years ago [37]. While rhBMP-2 is expensive, especially in the high dose used with Infuse, early predictive economic models suggested that the reduced operating times, hospital stays, and complication rates provided by Infuse might completely offset the large initial costs of rhBMP-2, resulting in roughly equivalent costs for Infuse and autograft [91,92]. Some studies have evaluated actual cost data to further explore cost differences, and results are shown below. These numbers are intended only to provide estimates of Infuse’s cost compared with alternative treatments, as cost varies by study and by patient. Notably, rhBMP-2′s expense limits its clinical applicability, regardless of comparison with alternate treatments [93].

A cost-comparing study of 102 spinal fusion patients over sixty years of age included 52 patients treated with iliac crest autograft and 50 treated with Infuse. Without factoring in complications and additional spine treatments, this study reported an average cost of USD 34,235 for the autograft group and USD 36,530 for the Infuse group, suggesting that the substantial upfront cost of rhBMP-2 outweighed the increased operating time and hospital stay seen in autograft patients. However, after considering complications and surgical revisions, which occurred at a greater rate in the autograft group, the average cost for autograft treatment was USD 42,286, while Infuse treatment cost an average of USD 39,967 [94]. This study concluded that Infuse lowered overall costs because of higher treatment success rates, though costs were relatively similar between treatment groups.

Infuse’s cost has also been evaluated in open tibial fractures. In the METRC study discussed above, later complications and additional treatments were not considered in the cost analysis. The average cost for Infuse/allograft treatment was USD 14,155, while the average autograft treatment cost was USD 9086 [27]. This discrepancy may be slightly due to allograft costs of USD 13.25 per cm^3^, as the Infuse/allograft patients received up to 60 cm3 of allograft, resulting in an upper limit of USD 795 per patient spent on allograft. However, the study reported an average cost of USD 5400 per Infuse package, suggesting that the major reason for the cost increase in the Infuse/allograft group was the rhBMP-2 product. While this analysis did not factor in costs of later complications and revisions, it reported increased healing and reduced complications for autograft patients, suggesting that the final cost difference was likely even more favorable toward autograft than the roughly USD 5000 difference reported [27].The BESTT and Jones studies, which supported the tibial application of Infuse, did not include a cost analysis, though the BESTT researchers suggested that the clinical benefits of Infuse would lower overall medical costs [73,76].

In maxillofacial applications, cost reports are scarce for on-label applications [95], but existing reports seem to follow the trend of Infuse reducing overall costs when clinical outcomes are improved in the Infuse group. In one study of off-label use of Infuse in alveolar cleft reconstruction, costs for demineralized bone plus Infuse treatment averaged USD 4836, while autograft treatment cost an average of USD 6892, though as expected, the surgical material cost was higher in the Infuse group [96]. Another study reported an average total cost of USD 21,800 for autograft in alveolar cleft reconstruction for older patients, and an average total of USD 11,100 for the Infuse group [97]. In these applications, Infuse may be the more cost-efficient option, but there is a lack of data necessary to make that definite conclusion, especially regarding on-label maxillofacial use.

In summary, Infuse seems to reduce overall costs or improve clinical outcomes compared to pre-existing treatments. However, as it is also summarized in Table 3, which outlines the FDA- and EMA-approved clinical applications of Infuse Bone Graft (U.S.) and InductOs (EU) together with their advantages, disadvantages, and cost considerations, this is not the case in every study. Despite its many benefits, Infuse has not significantly decreased treatment costs, which can be further investigated in future studies.

## 6. Future Perspectives

Breakthroughs in bone regeneration technologies are reshaping the landscape of regenerative medicine, driving the development of increasingly biocompatible, effective, and targeted strategies for bone repair across diverse clinical fields. Recombinant growth factor–based approaches, in particular, have played a pivotal role in expanding available therapeutic options. While Infuse^®^ Bone Graft has already revolutionized the field of bone tissue engineering through its potent osteoinductive properties and widespread adoption in spinal fusion procedures and other clinical applications, the future of this technology lies in strategic enhancement—not replacement. Optimizing delivery vectors, incorporating synergistic biological cues, and addressing the neurovascular demands of vertebral bone healing represent key frontiers for next-generation applications.

### 6.1. Spinal Fusion

In recent spinal fusion applications, transforaminal lumbar interbody fusion (TLIF) procedures using rhBMP-2 have achieved remarkably high fusion rates. A 2024 multicenter Korean trial utilizing E. coli-derived rhBMP-2 in 30 patients reported 100% fusion at both 1- and 2-year follow-ups, accompanied by significant improvements in Oswestry Disability Index (ODI), SF-36, and VAS pain scores, with no incidence of seroma, radiculitis, heterotopic ossification, cage migration, or neurological deficit. Similarly, a retrospective 2024 analysis demonstrated that rhBMP-2 accelerated time-to-fusion by approximately 50%, regardless of patient age or bone quality. Comparative studies indicate that rhBMP-2 provides 10–12% higher fusion rates than iliac crest autograft at 24 months and yields an average ODI improvement of 3.5 points, with only a modest elevation in early postoperative pain risk (OR ≈ 1.8) [49].

To mitigate known dose-related complications—including ectopic bone formation, osteolysis, radiculitis, and retrograde ejaculation—emerging strategies have focused on low-dose delivery platforms using hydrogels, nanogels, or hydroxyapatite/β-TCP composites. These systems have demonstrated a comparable fusion efficacy, with significantly reduced inflammation in ovine and porcine models [98,99,100,101,102]. In infectious spinal pathologies such as pyogenic spondylodiscitis or osteomyelitis, rhBMP-2 combined with surgical debridement and antibiotic therapy achieved a 97.8% fusion, 68% neurological improvement, and only 1.5% recurrence of infection, supporting its safe use in compromised settings [102,103,104]. Collectively, these outcomes underscore the robust osteogenic performance of rhBMP-2 and highlight the potential of controlled-release bioactive scaffolds in further enhancing clinical results.

Yet, the next leap in spinal regeneration may come not only from enhanced bone fusion but from simultaneous neural regeneration. Beyond structural osseointegration, vertebral bone repair inherently requires the reconstitution of the neurovascular microenvironment within the osteon and Haversian canal system. These cylindrical cortical bone units contain CGRP^+^ sensory and TH^+^ sympathetic fibers, both of which are emerging as pivotal regulators of osteogenesis and nociception [105,106,107,108,109,110,111]. The Haversian canal functions not just as a conduit for nutrient delivery but also as a hub of neurogenic signaling, essential for osteoblast activation, mechanical adaptation, and homeostasis [112,113,114,115].

Disruption of these neurovascular elements—whether via fusion procedures, trauma, or degenerative disease—leads to impaired remodeling, sensory deficits, and chronic pain, creating an urgent therapeutic gap. The presence of neuropeptides like substance P and CGRP in cortical bone, and the inhibitory role of β-adrenergic sympathetic input on osteoblasts, suggest a bidirectional neuroskeletal axis [116]. Animal models of denervation consistently demonstrate delayed fracture healing, reduced callus formation, and altered remodeling kinetics, confirming the centrality of neural inputs in bone repair [107,108,109,110,111]. Although Infuse is not indicated for neural regeneration, its active component, recombinant human BMP-2 (rhBMP-2), has demonstrated neurotrophic and neuroprotective effects. In vitro, rhBMP-2 (~50 ng/mL) stimulates neurite outgrowth by 1.3–1.5× in SH-SY5Y dopaminergic cells and protects against MPP^+^ and 6-OHDA toxicity [49,50,51,117,118,119]. In vivo, BMP-2 enhances dopaminergic neuron survival and improves motor function in lesioned rodent models [120]. It also promotes the differentiation of enteric nitrergic and catecholaminergic neurons via Smad1/5/8 signaling, while modulating glial behavior and axonal repair through cytoskeletal and epigenetic pathways [49,50,51,117,118,119].

Translational efforts are now focusing on hybrid scaffold platforms that co-deliver low-dose rhBMP-2 with neural guidance cues—such as laminin-coated nanofibers, magnetic nanoparticles, or engineered exosomes containing BMP-2 mRNA. These biofunctional constructs aim to re-establish both the osteogenic and neurogenic microenvironment within the vertebra. For example, aligned nanofiber–hydrogel composites can guide axonal pathfinding while promoting osteoblast adhesion, and magnetically responsive scaffolds enable directional control of neural regeneration via external fields. Exosome-based BMP-2 vectors offer prolonged release, immunomodulation, and compatibility with minimally invasive delivery methods—potentially overcoming current limitations of systemic BMP exposure. While still in the preclinical stage, these dual-regeneration constructs represent a paradigm shift in spinal tissue engineering. By targeting both structural repair and neurovascular integration, they offer the possibility of restoring full Haversian function—combining mechanical strength, biological adaptability, and sensory performance. As material science, stem cell biology, and neuroengineering converge, the clinical translation of such strategies may redefine spinal fusion not merely as a mechanical fix but as a comprehensive tissue restoration procedure [98,99,101,104,121,122].

Although still at the preclinical stage, dual-regeneration strategies (targeting both bone and neural tissue) represent a paradigm shift in spinal tissue engineering. By simultaneously promoting structural repair and restoring the neurovascular microenvironment, they offer the potential to fully reconstitute Haversian canal function—combining mechanical strength, biological adaptability, and sensory performance. Preclinical models have explored a diverse range of platforms: aligned nanofiber–hydrogel scaffolds that guide axonal growth while supporting osteogenesis through co-delivery of BMP-2 [117]; electrospun fibrous scaffolds (e.g., PCL/HA or PLGA/HA) providing a sustained BMP-2 release to mimic the extracellular matrix and enhance the neurogenic niche [123]; magnetic nanoparticles that direct neurite extension under external magnetic fields, combined with BMP-2 for hybrid osteo/neuroinduction [124]; BMP-2 mRNA-loaded exosome hydrogels enabling prolonged local expression and potential neuroimmunomodulatory effects [125]; and functionalized nanofibers and composite scaffolds incorporating exosomes or magnetic elements for targeted, multimodal regeneration [126].

The comparative table below (Table 4) summarizes key preclinical approaches for neural tissue regeneration alongside Infuse/InductOs (rhBMP-2) in spinal fusion contexts, followed by a detailed interpretation and evidence-based discussion.

In summary, while Infuse remains an osteogenically dominant therapy, its underexplored neuroregenerative potential, together with an improved understanding of the Haversian neurovascular niche, provides a compelling rationale for multifunctional biomaterial systems. Advances in controlled release, bioactive surface engineering, and smart nanotechnologies will be critical to translating these discoveries into safer, more effective spinal interventions—ushering in an era where fusion means not just the union of bone but functional neural recovery.

### 6.2. Open Tibial Fracture

Although clinical translation of rhBMP-2 delivered via absorbable collagen sponge (ACS) in open tibial fractures has highlighted significant benefits, conventional collagen scaffolds suffer from rapid degradation and burst release, complicating dose control and raising inflammation and ectopic bone risks. To address these shortcomings, recent studies have engineered collagen composites that not only improve mechanical strength, but also enable sustained, programmable release of BMP-2.

Specifically, in one study, a b-layered collagen/collagen-Mg-hydroxyapatite scaffold with absorbed BMP-2 showed sustained growth factor retention and enhanced bone formation in preclinical models [127]. Likewise, innovations in heparin-modified or chemically cross-linked collagen hydrogels have achieved a low-dose BMP-2 release sustained over 28 days, improving both safety and bone defect healing in rodent models [128].

However, other emerging delivery systems—such as hydroxyapatite/β-TCP composites and nanogel-based hydrogels—are also enabling programmable, sustained release of rhBMP-2. These platforms have shown improved bone union rates in pre-clinical long-bone models while significantly reducing inflammatory responses. Furthermore, they support immune homeostasis and exhibit microbial resistance, making them particularly advantageous in contaminated open fractures [129]. Lastly, the arrival of stimulus-responsive biomaterials, capable of adjusting their drug release profiles in response to environmental cues, indicates a new era of fracture-specific dosing strategies, tailoring BMP release to the distinct phases of bone healing [130].

### 6.3. Maxillary Augmentation

Dental implants have revolutionized restorative dentistry, offering a durable and aesthetically pleasing solution for replacing missing teeth. The foundation of modern implantology was established by Dr. Per-Ingvar Branemark in the 1960s, with the introduction of the osseointegration concept, and since then, continuous advancements in implant materials, surgical protocols, and prosthetic technologies have significantly enhanced patient clinical outcomes. One of the most critical procedures in implant dentistry is sinus floor augmentation, which aims to restore sufficient vertical bone height for implant placement. However, the augmented region is susceptible to changes in maxillary sinus air pressure, making volumetric stability of the grafted material a key determinant of long-term success. Ideal graft materials should exhibit controlled resorption, maintaining graft height during early healing while supporting new bone formation [131,132].

Bone graft consolidation hinges on the progressive replacement of the graft by vital bone through functional remodeling. This dynamic process is influenced by systemic factors (such as age, underlying health conditions, and medications) and local anatomical characteristics, including sinus morphology [133]. While the clinical use of Infuse^®^ Bone Graft in sinus floor and alveolar ridge augmentation has demonstrated high success rates with reduced donor site morbidity, the continued incidence of dose-dependent complications—most notably edema and localized inflammation—underscores the need for next-generation delivery platforms.

Ongoing research is focused on enhancing guided tissue regeneration (GTR) and guided bone regeneration (GBR) through biomaterials that offer a smarter, more adaptive therapeutic delivery. A promising future direction involves the development of stimuli-responsive scaffolds capable of spatially and temporally regulated release of rhBMP-2 and other growth factors [134]. For instance, nanoporous collagen matrices and engineered hydrogel systems can reduce burst release, enabling sustained delivery at therapeutically effective, yet lower, doses.

Concurrently, synthetic biomaterials and resorbable biopolymers, such as polyethylene glycol (PEG), designed to replicate key biological properties of bone have become central to regenerative strategies. Additionally, injectable albumin platelet-rich fibrin (Alb-PRF/e-PRF) has shown enhanced stability and resistance to degradation, suggesting its future potential as a substitute for collagen membranes [131,134,135]. Moreover, minimally invasive approaches—including 3D-printed or injectable scaffolds tailored to individual anatomical variations—hold the potential to reduce surgical morbidity and improve precision in graft placement [134,135].

Importantly, mechanical loading plays a crucial role in peri-implant bone formation, mineralization, and remodeling. Stacchi et al. highlighted the impact of mechanical stimuli during the healing phase, with improper or premature loading potentially impairing osseointegration, particularly in sinus-augmented regions. Defining optimal loading conditions remains a challenge but is vital for improving long-term implant stability and function [133].

Beyond materials science, epigenetic strategies are also gaining traction in the field. By modulating gene expression profiles relevant to osteogenesis, these approaches may enhance the regenerative capacity of host tissues and complement conventional bone graft techniques [134].

Taken together, these advancements could establish a new standard of care in craniofacial bone regeneration—one that preserves the clinical benefits of rhBMP-2 while overcoming its current biological and regulatory limitations. However, while many of these innovations have demonstrated strong performance in in vitro or preclinical models, challenges remain regarding high manufacturing costs, complexity of clinical handling, and large-scale feasibility. Robust clinical studies are needed to assess long-term implant integration, graft stability, and the incidence of rare but serious complications. Overcoming these barriers is essential to translate laboratory success into real-world therapeutic impact.

## 7. Conclusions

The Infuse^®^ Bone Graft has played a pivotal role in redefining the clinical landscape of bone regeneration, representing the first widely adopted osteoinductive bone graft substitute with regulatory approval for multiple indications. Its introduction demonstrated how growth factor-based technologies can expand therapeutic options while reducing the need for autografts. Its ability to eliminate donor-site morbidity, simplify surgical protocols, and achieve reliable fusion rates in select applications, such as anterior lumbar interbody fusion (ALIF), open tibial fractures, and maxillofacial augmentation, highlights its potential as a viable alternative to autografts.

Nonetheless, Infuse^®^ is not without significant limitations. The high cost of rhBMP-2 remains a prohibitive factor for routine use, particularly in resource-constrained settings or procedures with uncertain cost–benefit margins. Moreover, the product’s suboptimal release kinetics—characterized by an initial burst release—has been implicated in a range of adverse events, including ectopic bone formation, soft tissue swelling, inflammation, and osteolysis. These risks have been especially prominent in off-label applications and underscore the need for careful patient selection, appropriate dosing, and strict adherence to regulatory guidelines to maximize benefits while minimizing risks.

At the tissue level, Infuse^®^ consistently demonstrates strong osteoinductive effects, promoting mesenchymal stem cell recruitment, vascularization, and organized bone remodeling. However, its clinical success is tightly coupled with the performance of its collagen-based scaffold carrier, which lacks mechanical strength and provides limited control over growth factor retention. While Infuse^®^ represents a substantial technological advance over purely osteoconductive materials, it also illustrates the inherent trade-offs between biological potency and safety when using potent growth factors like rhBMP-2. Integrating these strategies could lead to more predictable outcomes and broaden the clinical applications of controlled bone regeneration technologies.

Looking forward, the limitations of Infuse^®^ are spurring innovation in scaffold design, delivery systems, and biomolecular engineering. Future bone graft technologies are likely to incorporate tunable, multi-functional carriers, controlled delivery of multiple signaling molecules, and patient-specific scaffolds fabricated via advanced manufacturing techniques. Additionally, emerging alternatives—such as osteoinductive small molecules, gene therapy, and immunomodulatory approaches—may eventually supplement or supplant protein-based therapies like rhBMP-2.

In conclusion, while Infuse^®^ has set a new benchmark in synthetic bone grafting, its clinical adoption offers important lessons for the next generation of regenerative solutions: harness new biological knowledge in order to balance biological potency with safety, ensure cost-effectiveness, and match the technology to well-defined clinical needs. A nuanced, evidence-based approach to its use, combined with ongoing interdisciplinary innovation, will be essential to usher in the next generation of regenerative solutions for orthopedic and craniofacial applications. Continued research in this direction holds the promise of making advanced bone regeneration accessible, safe, and effective for a wider patient population.

## Figures and Tables

**Table 1:** Advantages (compared with autograft) and disadvantages of Infuse in lumbar spinal fusion [18,25,26,37,57,58].

Advantages vs. Autograft	Disadvantages (Side Effects)
No tissue harvesting surgery required	High rates of ectopic bone growth (typically between 33% to 70%)
~8% improved fusion rate	29% rate of radiculitis
Lower reoperation rate	19% rate of dural tear
Reduced blood loss	5% rate of hematoma/seroma
Reduced patient-reported pain	Reported cases of Vertebrae osteolysis, Device subsidence and dislodgement, Soft tissue swelling and inflammation, Cervical swelling-related death, Connection to cancer, Retrograde ejaculation, Wound dehiscence, Increased infection, Increased neurologic deficits, Arachnoiditis
Better patient-reported overall health score	
Shorter surgery time	
Shorter hospital stay	
Faster return to work	

**Table 2:** Adverse events associated with rhBMP-2 in spinal fusion: harmonized definitions, reported incidence ranges with primary sources, and juxtaposition with absolute fusion-rate gains at ≈24 months compared with iliac crest bone graft (ICBG) [18,25,26,37,57,58,59,60,61,62,63,64,65].

Adverse Event (Harmonized Definition)	Rate/Range (Study Context)	Follow-Up Window	Key References	Typical Absolute Fusion Gain vs. Control (~24 Months)
Heterotopic/ectopic bone (radiographic, CT-detected)	13–75% radiographic; symptomatic far lower	6–12 months CT	[26,57,58,60]	n/a
Heterotopic/ectopic bone (symptomatic, requiring treatment/revision)	0–2%	6–24 months	[26,57,61]	n/a
Radiculitis (new/worsened radicular pain)	7–14% (posterior fusions)	Early postop—3 months	[57,61]	n/a
Osteolysis/osteoclastic resorption (adjacent to cage, CT)	5–20% (series-dependent)	3–12 months CT	[57,60,62]	n/a
Seroma/hematoma (wound collections)	1–3%	30–90 days	[18,57,61]	n/a
Wound infection/dehiscence	2–4%	30–90 days	[18,57,61]	n/a
Urinary retention	2–8%	In—hospital–30 days	[57,61]	n/a
Ileus	1–3%	In—hospital–30 days	[57,61]	n/a
Respiratory complications (pneumonia, aspiration)	0.5–1%	In—hospital–30 days	[57,61]	n/a
Incidental durotomy (approach-related, not BMP-specific)	3–10% (higher in revisions; 1.8–6.2% MIS-TLIF)	Intra-op	[58,63,64,65]	n/a
Retrograde ejaculation (ALIF, male)	2–8% overall; 7.2% rhBMP-2 vs. 0.6% control (L5–S1 RCT)	3–24 months	[37,57,58]	ALIF: +6–16% vs. ICBG ([25,37])
Fusion efficacy (PLF/PLIF/TLIF)	–	–	[18,25,37]	PLF/PLIF/TLIF: +5–10% vs. ICBG

Abbreviations: ICBG, Iliac Crest Bone Graft; PLF, Posterolateral Fusion; PLIF, Posterior Lumbar Interbody Fusion; TLIF, Transforaminal Lumbar Interbody Fusion; ALIF, Anterior Lumbar Interbody Fusion; CT, Computed Tomography; MIS, Minimally Invasive Surgery.

**Table 3:** Summary of FDA- and EMA-approved clinical applications of Infuse Bone Graft and InductOs, respectively [25,26,27,42,52,54,73,90,91,92,93,94,95].

Clinical Application	Spinal (Lumbar) Fusion	Open Tibial Fracture	Maxillary Augmentation
FDA Approval Year:	2002	2004	2007
EMA Approval Year:	2002	2002	-
Major Advantage:	High vertebral fusion rate without harvest surgery	Higher bone formation rate compared to no grafting	High bone formation rate without harvest surgery
Major Disadvantage:	Many concerning side effects	Lack of data, possible regeneration inferiority compared to autograft	Some side effects
Cost Per Package:	USD 2500–6000		
Total Cost Relative to Autograft Alternative:	Likely comparable, but definitive conclusion is difficult. Cost is related to clinical success.		

**Table 4:** Comparative neural regeneration strategies using rhBMP-2 in spinal fusion [117,123,124,125,126].

Approach	Key Mechanism	BMP-2 Role	Evidence and Findings
Aligned nanofiber and hydrogel scaffolds	Laminin-coated electrospun fibers guide axon growth within a hydrogel matrix	Co-delivered with BMP 2 to support osteogenesis and neural guidance	Guides neurite outgrowth significantly; enhanced neurite tracking in laminin-functionalized hydrogel
BMP 2-loaded nanofibers (e.g., PCL/HA)	Fibrous scaffold mimicking ECM with sustained BMP 2 release	Encourages bone formation; may support neurogenic niche	PLGA/HA fibrous scaffolds with BMP 2 mimic ECM, though neural data limited
Magnetic nanoparticles (MNPs)	MNPs guide neurite extension via external magnetic fields	Can be co-functionalized with BMP 2 to hybridize osteo/neuroinduction	Promising concept in SCI repair; shown neurite extension under guidance
BMP 2-mRNA exosome hydrogels	Exosomes deliver BMP 2 mRNA, enabling sustained release from host cells	Supports osteogenesis; hypothesized to exert neuroimmunomodulation	Engineered BMP 2 exosomes in hydrogel accelerated bone repair
Nanofiber–hydrogel composite for SCI	Composite scaffolds bridge injury sites and support axon regrowth	Could be adapted to BMP 2 delivery for combined therapy	Improved neural repair after spinal cord contusion in rats
Exosome-functionalized MOF scaffolds	Exosome-coated magnesium scaffolds drive osteogenesis/angiogenesis	Platform could be repurposed for targeted BMP 2 neurodelivery	Enhanced osteogenic and angiogenic markers observed

## Data Availability

No new data were created or analyzed in this study. Data sharing is not applicable to this article.

## Abbreviations

The following abbreviations are used in this manuscript:

rhBMP-2	Recombinant human BMP-2
ECM	Extracellular Matrix
ACS	Absorbable Collagen Sponge
TLIF	Transforaminal Lumbar Interbody Fusion
BESTT	BMP-2 Evaluation in Surgery for Tibial Trauma
METRC	Major Extremity Trauma Research Center
ODI	Oswestry Disability Index
GTR	Guided Tissue Regeneration
GBR	Guided Bone Regeneration
VEGF	Vascular endothelial growth factor
HO	Heterotopic Ossification
ICBG	Iliac Crest Bone Graft
PLF	Posterolateral Fusion
PLIF	Posterior Lumbar Interbody Fusion
ALIF	Anterior Lumbar Interbody Fusion
CT	Computed Tomography
MIS	Minimally Invasive Surgery
